# Gγ and Gα Identity Dictate a G-Protein Heterotrimer Plasma Membrane Targeting

**DOI:** 10.3390/cells8101246

**Published:** 2019-10-13

**Authors:** Paweł Mystek, Beata Rysiewicz, Jan Gregrowicz, Marta Dziedzicka-Wasylewska, Agnieszka Polit

**Affiliations:** Department of Physical Biochemistry, Faculty of Biochemistry Biophysics and Biotechnology, Jagiellonian University, Gronostajowa 7, 30-387 Kraków, Poland; pawel.mystek@uj.edu.pl (P.M.); beata.rysiewicz@doctoral.uj.edu.pl (B.R.); jangregrowicz@caltech.edu (J.G.); marta.dziedzicka-wasylewska@uj.edu.pl (M.D.-W.)

**Keywords:** protein-membrane interaction, spatiotemporal protein dynamics, G-proteins, FLIM-FRET, FRAP

## Abstract

Heterotrimeric G-proteins along with G-protein-coupled receptors (GPCRs) regulate many biochemical functions by relaying the information from the plasma membrane to the inside of the cell. The lipid modifications of Gα and Gγ subunits, together with the charged regions on the membrane interaction surface, provide a peculiar pattern for various heterotrimeric complexes. In a previous study, we found that Gαs and Gαi_3_ prefer different types of membrane-anchor and subclass-specific lipid domains. In the present report, we examine the role of distinct Gγ subunits in the membrane localization and spatiotemporal dynamics of Gαs and Gαi_3_ heterotrimers. We characterized lateral diffusion and G-protein subunit interactions in living cells using fluorescence recovery after photobleaching (FRAP) microscopy and fluorescence resonance energy transfer (FRET) detected by fluorescence lifetime imaging microscopy (FLIM), respectively. The interaction of Gγ subunits with specific lipids was confirmed, and thus the modulation of heterotrimeric G-protein localization. However, the Gα subunit also modulates trimer localization, and so the membrane distribution of heterotrimeric G-proteins is not dependent on Gγ only.

## 1. Introduction

G-protein-coupled receptor (GPCR) stimulation results in the activation of a Gα subunit and a Gβγ complex of the heterotrimeric G-protein. Both the activated components of the heterotrimer modulate the function of downstream effector proteins located on the cytosolic surface of the cell membrane. The intracellular response effect is caused by second messenger molecules after the stimulation of effector proteins such as adenylyl cyclase, phospholipase C-β, and ion channels. There are 18 Gα, five Gβ, and 12 Gγ genes in the human genome [1]. The Gα subunit family is the most diverse among G-proteins and is responsible for the specificity of interactions with GPCRs [2]. There is evidence of distinct activity of the Gβγ dimer in terms of G-protein activation and modulation of effector proteins [3].

Several different lipids are covalently attached to Gα and Gγ subunits, and as hydrophobic anchors, lipids promote localization to cellular membranes. Apart from the subunits Gα_t_ and Gα_gust_, all Gα subunits are palmitoylated, and some are also myristylated. Palmitoylation is a post-translational modification that involves the addition of saturated 16-carbon palmitic acid to a specific cysteine in an amino acid sequence through a thioester bond (S-palmitoylation). This modification is unique among lipidations and can be quickly reversed under in vivo conditions [4,5]. It is also possible to attach palmitoleic acid by an amide bond to the glycine residue in the N-terminal fragment of the Gαs protein (N-palmitoylation) [6]. Myristylation is a co-translation or post-translation modification which consists of attaching the myristate (a 14-carbon saturated fatty acid) to the N-terminal glycine residue through a peptide bond [7]. The non-myristylated Gα subunits (Gαs, Gαq, and Gα_12/13_ families) contain the motif of basic residues, arginines and lysines, forming a charged surface on one side of the N-terminal helix [8,9,10,11]. Such a charged protein area is able to interact with negatively-charged lipid head groups. A positively charged surface is also present in the Gβ structure. Such a region, through electrostatic interactions favors binding to acidic membrane phospholipids [12].

All Gγ subunits undergo post-translational isoprenylation. Prenylation is an irreversible multistage modification that involves the transfer of a C_15_ farnesyl or C_20_ geranylgeranyl group to a cysteine residue within the C-terminal CaaX motif via a thioether bond. Farnesylation occurs in the Gγ_1_, Gγ_9_, and Gγ_11_ subunits; the other subunits of Gγ are geranylgeranylated [13]. According to the two-signal hypothesis or kinetic trapping [14,15,16,17,18] a single membrane binding signal might be insufficient for proper membrane docking. However, a combination of two or more signals such as lipid anchor, poly-basic sequence or other motifs can ensure high-affinity interaction with cellular membranes.

Apart from differences in the length of anchors, the literature distinguishes three groups of gamma subunits based on transient translocation to endomembranes. The γ_1_, γ_11_, γ_9_, and γ_13_ belong to a group characterized by rapid translocation, γ_5_ and γ_10_ translocate slowly, while the other Gγ subunits, previously identified as non-translocating, are currently described as the slowest translocating [19,20]. It is noteworthy, that the translocation process is acylation/deacylation cycle dependent and the activation of G-protein is not necessary [21]. It is currently accepted that the factors that influence the behavior of activated G-proteins are not only heterotrimer composition and lipid modification but also local membrane environment, receptor coupling, the presence or absence of effector proteins or accessibility of other interacting partners.

In previous reports, we showed a separate location of heterotrimers of Gαs and Gαi_3_ subunits with Gβ_1_γ_2_ complex in HEK 293 cells [22]. Here we examined the effect of distinct Gγ subunits, Gγ_2_ and Gγ_9_, on heterotrimer behavior prior to receptor activation. The combined approach of two fluorescence microscopy techniques was used, fluorescence lifetime imaging microscopy–fluorescence resonance energy transfer (FLIM–FRET) technique to examine protein-protein interactions and the fluorescence recovery after photobleaching (FRAP) technique to monitor membrane dynamics of complexes. We confirmed the interaction of the Gγ subunit with specific lipids and thus the modulation of heterotrimeric G-protein localization. However, the membrane distribution of heterotrimeric G-proteins is not only Gγ dependent. Results presented herein indicate that the Gα subunit also modulates trimer localization and the nature of that impact is to some extent similar to that of Gγ. Therefore, the role of other subunits of G-protein trimer partitioning process appears to be significant.

## 2. Materials and Methods

### 2.1. Chemicals and Protein Constructs

All chemicals were purchased from Sigma (Sigma Aldrich, Poznań, Poland) unless otherwise indicated. All DNA sequences of Gα, β, and γ subunits in pcDNA3.1+ used were purchased from UMR cDNA Resource Center (Bloomsberg, PA, USA). Plasmids pEYFP–N1, pEGFP–N1, and pmCherry–N1 were purchased at Clontech (Mountain View, CA, USA). The fusion proteins of the Gα subunits with fluorescent proteins (FP) was performed as described earlier [22]. In the Gαs_IEK_ chimeric protein, the conserved region Ile_26_–Glu_27_–Lys_28_ (G.HN.43–45; (I/L)-(E/D)-(K/R) motif in Gα family) was modified into a sequence of three alanine residues. These mutations were carried out using the Quick Change method and resulted in the elimination of the interaction of the mutant Gαs_IEK_ protein with the Gβγ dimer [23]. The restriction-free cloning method [24] was used to modify the Gβ_1_ subunit. As a first step, the FP sequence was multiplied and cloned at the N-terminus of the Gβ_1_ subunit and this construct was used in the FLIM-FRET experiments. In the next PCR, a His-tag sequence was added at the N-terminus of the FP. This modification enabled carrying out pull-down experiments. All constructs were checked by determining the nucleotide sequence.

### 2.2. Cell Culture

The HEK 293 cells (American Type Culture Collection, Manassas, VA, USA) were cultivated and transiently transfected as described earlier [22]. The cells were transfected with 0.15–0.6 μg of DNA/dish and the ratio of the DNA coding of the Gα, Gβ, and Gγ subunits was 2:3:3. For the pull-down assay, cells were cultured on a 100 mm diameter dish and the amount of DNA for transfection was increased accordingly.

### 2.3. Determination of Intercellular cAMP Concentration

Changes in cAMP concentration were determined by using the cAMP ELISA chemiluminescent kit (STA-500, Cell Biolabs Inc, San Diego, CA, USA). Cells were seeded onto six-well plates and 24 h later transfected with 1.85 μg plasmid DNA/well. HEK 293 cells were co-transfected with the D_2_R dopamine receptor in the case of Gαi_3_ or adenosine A_2A_R receptor in the case of Gαs and Gαs_IEK_. Three days after transfection the cells were treated for 10 min with 1 μM sumanirole maleate in minimum essential medium (MEM) containing 0.5% fetal bovine serum (FBS) and phosphodiesterase inhibitors 0.5 mM 3-isobutyl-1-methylxanthine (IBMX) and 0.1 mM 4-(3-butoxy-4-methoxybenzyl)imidazolidin-2-one (Ro 20–1724) or for 15 min with 1 μM 2–phenylaminoadenosine in MEM medium containing 0.5% FBS. Non-transfected cells were used as controls. After stimulation, the concentration of cAMP in harvested cell lysates was determined according to the manufacturer’s instruction. The chemiluminescent signal was measured by using a Synergy H1 microplate reader (BioTek Instruments, Winooski, VT, USA). To minimize batch-to-batch variations, in each experiment the signals were normalized to the average signal in the control group (non-transfected cells), and the normalized data were summarized across all experiments.

### 2.4. Pull-Down Assay

Interaction of Gαs or Gαs_IEK_ mutants with the Gβ_1_ or Gβ_1_γ_2_ dimers was studied by pull-down on a nickel-charged affinity beads (NiNTA agarose). HEK 293 cells, transiently expressing bait His-tagged Gβ_1_-mCherry alone or full heterotrimeric Gαsβ_1_γ_2_ or Gαs_IEK_β_1_γ_2_ complexes, were lysed in ice-cold lysis buffer (50 mM Tris, pH 8.0, 5 mM MgCl_2_, 10 mM β-mercaptoethanol, 100 mM NaCl, 20 μM GDP, benzonase, inhibitors). After sonication and centrifugation (50,000× *g*, 45 min, 4 °C), the supernatant (cytoplasmic fraction) containing His_6_-tagged mCherry-Gβ_1_ was loaded onto the NiNTA resin and washed with wash buffer (lysis buffer with 50 µM guanosine-5′-triphosphate, GTP, and without benzonase and inhibitors) and utilized for studying interactions with Gαs. The membrane fractions containing membrane-bound Gαsβ_1_γ_2_ or Gαs_IEK_β_1_γ_2_ heterotrimers were suspended in wash buffer supplemented with 1% n-dodecyl-β-D-maltopyranoside, DDM (Anatrace, Maumee, OH, USA) and homogenized (Teflon–glass homogenizer, Sigma Aldrich, Poznań, Poland). After overnight solubilization (4 °C), clarified membrane fractions (50,000× *g*, 45 min, 4 °C) were incubated for 3 h at 4 °C with NiNTA resin.

In the next step, lysate from cells expressing Gαs or Gαs_IEK_, after sonication and centrifugation (cytoplasmic fraction), were incubated for 3 h at 4 °C with His-Gβ_1_-trapped NiNTA. The membrane-bound Gαs or Gαs_IEK_ were isolated in the same way as full heterotrimers, followed by incubation with His-Gβ_1_-trapped NiNTA. Complexes bound to the beads were isolated by centrifugation, washed three times with ice-cold lysis buffer and eluted in wash buffer containing 0.5 M imidazole. As negative control, a cell lysate without protein expression (negative cell lysate) was used. The eluted complexes were separated on 15% polyacrylamide-SDS gel and visualized by Western blot using antibodies against Gαs (Novus, Centennial, CO, USA), His-tag (Proteintech, Rosemont, IL, USA), and mCherry (Proteintech).

### 2.5. Confocal Microscopy

HEK 293 cells producing Gαs, Gαs_IEK_ or Gαi_3_ or full heterotrimeric complexes (Gαsβ_1_γ_2_, Gαsβ_1_γ_9_, Gαs_IEK_β_1_γ_2_, Gαs_IEK_β_1_γ_9_, Gαi_3_β_1_γ_2_, Gαi_3_β_1_γ_9_) were imaged with the TCS SP5 laser-scanning confocal microscope (Leica Microsystems, Mannheim, Germany) with a 63 × 1.4 numerical aperture (NA) oil-immersion objective at 37 °C. Green or yellow fluorescence (mGFP or Citrine FP) was detected at 495–570 nm with 488 nm excitation (argon ion laser) and red (mCherry) at 610–700 nm with 594 nm excitation (laser diode). All measurements were taken on living cells at 37 °C in an air-stream cube incubator. Before imaging, the culture medium was replaced with fresh Dulbecco’s modified Eagle medium/nutrient mixture F-12 (DMEM-F12) containing 2% FBS without phenol red.

### 2.6. Fluorescence Lifetime Imaging

A time-domain fluorescence lifetime imaging FLIM was performed with a confocal laser-scanning microscope (TCS SP5; Leica Microsystems, Mannheim) additionally equipped with a single-photon counting device with picosecond time resolution (PicoHarp 300, PicoQuant, Berlin, Germany). Details of the instrumentation are as described previously [22,25]. Images were recorded using the following settings: 63× oil-immersion objective with numerical aperture (NA) 1.4 at 37 °C with a frame size of 512 × 512 pixels and 470 nm laser in pulse mode at 40 MHz. Fluorescence was detected by a single-photon avalanche photodiode (τ-SPAD, PicoQuant) in a narrow range of 500–550 nm (band-pass filter). Citrine and mCherry fluorophores were used as FRET pairs. SPAD signals were analyzed with the SymPhoTime software (PicoQuant). The decay of Citrine intensity distribution was approximated in the subsequent fluorescence lifetime analysis by a bi-exponential decay model wherein we estimated four parameters—fluorescence lifetimes (*τ*) and relative abundances of the components of the donor molecules in the sample. FLIM images were generated using the SymPhoTime software (PicoQuant) by displaying pixel-wise average lifetimes in pseudo-colors. During the analysis, the instrument response function (IRF) was applied to obtain short lifetime components with a high accuracy. 

Reduction of fluorescence lifetime between donor-only and FRET samples were calculated from the means of donor-only and FRET samples, with inclusion of fractional standard errors. The FRET efficiency (*E*) was calculated based on the following equation:

*E* = 1 − *τ_da_*/*τ_d_*, (1)

where: *τ_da_* is the lifetime of donor in the presence of acceptor molecules, and *τ_d_* is the lifetime of the donor only [26]. The energy transfer was analyzed only at the plasma membrane.

### 2.7. FRAP Measurements

All experiments using the FRAP microscopy technique were performed and analyzed as described earlier [22]. Briefly, FRAP images were collected by using the Leica TCS SP5 confocal scanning microscope with LAS AF software and an immersion lens 63 × 1.4 NA. Experiments were performed on transiently transfected live cells of the HEK 293 line at 37 °C. Just before imaging, the culture medium was replaced with fresh DMEM-F12 medium without phenol red and enriched with 2% FBS serum. Data was collected for at least 100 s after the photobleaching impulse.

### 2.8. Statistical Analyses

The FRAP and FLIM-FRET data was collected for at least five independent experiments. The distribution of data was determined (normality by Shapiro–Wilks’ *W* test; additionally, shape of the distribution by skewness and kurtosis analysis). Data were presented as mean ± standard error of the mean (S.E.M.) and the unpaired *t*-test was performed when the data were normally distributed. The assumption of equality of variances was verified by Levene’s test. Otherwise, data were represented as median ± median absolute deviation (MAD) and the Mann–Whitney *U* test was executed. Outliers, whose presence was evaluated by the box plot method or Grubbs’s test, were excluded from statistical analysis. The number of samples in each experiment (*n*) and *p*-values are presented in figure legends and tables. Statistical analysis was performed with Statistica (data analysis software system), version 13 (TIBCO Software Inc., Palo Alto, CA, USA, 2017; http://statistica.io).

## 3. Results

### 3.1. Functionality of Fluorescently-Tagged G-Proteins

We have shown previously that stimulatory Gαs and inhibitory Gαi_3_ subunits are located in distinct types of the membrane domains, depending on their specific activation state [22,25]. In previous experiments we reported that co-transfection of Gαs or Gαi_3_ with the Gβ_1_γ_2_ dimer and the dopamine D_1_ receptor influences the membrane location of Gαs and, to a lesser extent, of the Gαi_3_ [25]. In their presence, Gα, complexed with Gβ_1_γ_2,_ relocates outside the liquid-ordered membrane domains. Because of the possibility that this result arose from the concurrent actions of the Gβ_1_γ_2_ dimer and the D_1_ receptor, in the present study we focused solely on the effect of Gβγ. More specifically, we addressed the question whether the localization of the heterotrimer at the plasma membrane was controlled only by the Gβγ dimer.

**Figure 1:**
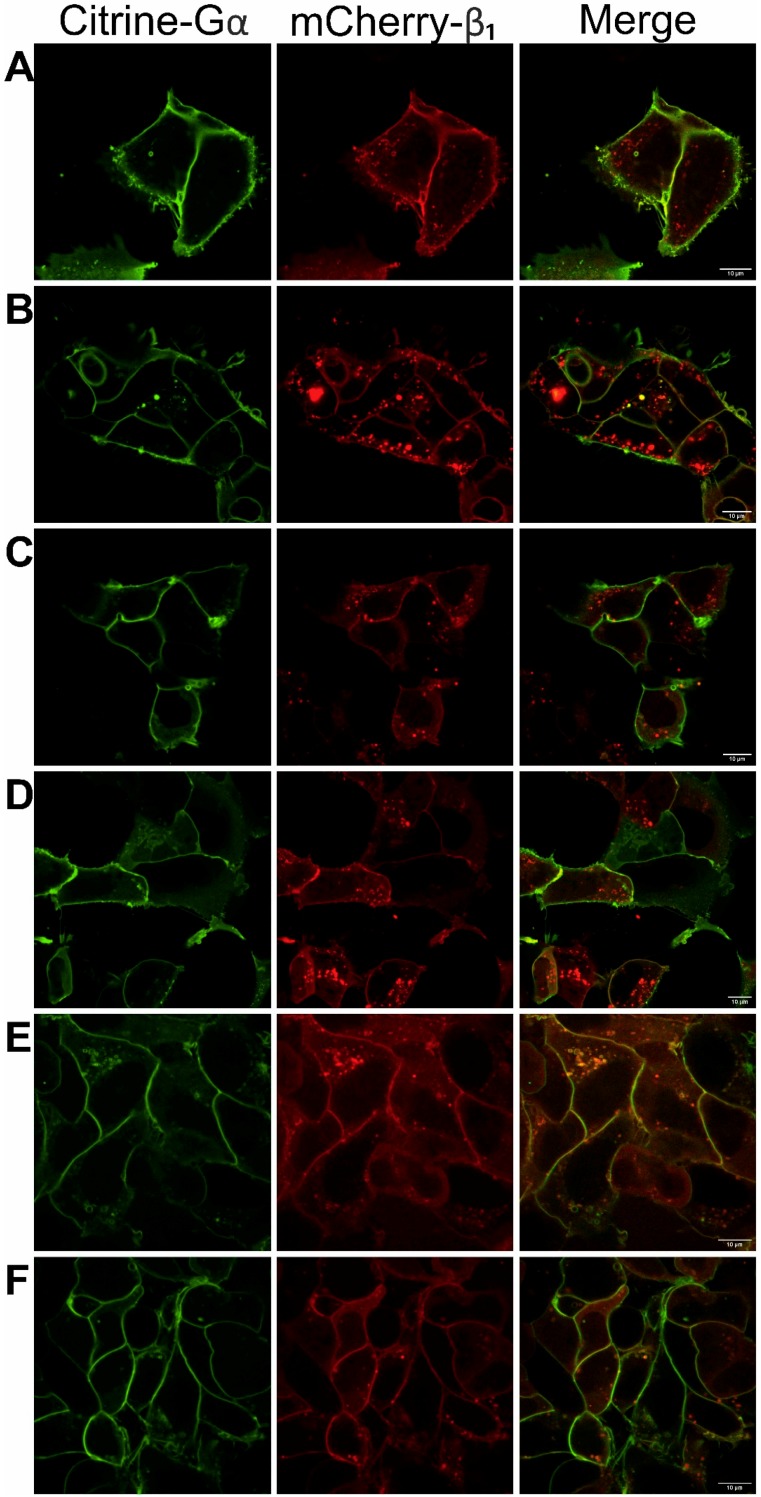
Cellular localization of Gαsβ_1_γ_2_, Gαsβ_1_γ_9,_ Gαi_3_β_1_γ_2_, and Gαi_3_β_1_γ_9_ heterotrimers. Representative confocal images of fluorescent protein (FP)-tagged Citrine–Gα subunits and mCherry–Gβ_1_γ dimers in transiently co-transfected HEK 293 cells. Localization of heterotrimers: (**A**) Gαsβ_1_γ_2_, (**B**) Gαsβ_1_γ_9_, (**C**) Gαs_iek_β_1_γ_2_, (**D**) Gαs_iek_β_1_γ_9_, (**E**) Gαi_3_β_1_γ_2_, (**F**) Gαi_3_β_1_γ_9_. Scale bar, 10 μm.

Tagging proteins of interest with FP might reduce their biological function due to undesired conformational changes or steric hindrance introduced by the tags. In order to avoid this, we selected loop-tagged Gαs and Gαi_3_ and amino-terminal-tagged Gβ_1_ for further optimization and analyses. The FP sequence was inserted into the L1 loop in the Gαs subunit (the loop from Glu_71_ to Ser_82_ residue (G.h1ha.7-G.h1ha.18) was exchanged), and into the second loop (αBC loop) within the helical domain of the Gαi_3_ subunit (FP sequence cloned after Ala_114_ residue (H.hbhc.2)). All investigated proteins displayed plasma membrane localization as shown by the fluorescence images, confirming that the presence of tags does not disturb their membrane-binding ability (Figure 1). The loop fusion Gαs has been reported to be functional [27]. Additionally, intracellular cAMP level was measured in order to check the functional activity of all designed fusion proteins.

The Gαs and Gαi_3_ subunits are distinct and provide the specificity for activation and inhibition, respectively, of adenylyl cyclase. Depending on the Gα subclass, HEK 293 cells were co-transfected with adenosine A_2A_ or the dopamine D_2_ receptor. In response to extracellular stimuli with a suitable agonist (2-phenylaminoadenosine or sumanirole), a rapid increase or reduction in the production of cAMP was detected for Gαs or Gαi_3_, respectively (Figure 1A,B). These results indicate that the tagged G-protein heterotrimers are signaling-active. The Gαs heterotrimers were capable of more than eight-fold induction in cAMP over the basal level. In turn, Gαi_3_ heterotrimers showed pronounced inhibitory effect. Gαi_3,_ tagged with Citrine or mGFP, was examined and no significant differences in cAMP production between these fusion proteins were found. Intriguingly, trimers composed of Gβ_1_γ_9_ changed the basal cAMP level in HEK 293 cells less efficiently than trimers consisting of Gβ_1_γ_2,_ especially Gαi_3_β_1_γ_9_. This finding may be explained by a potentially less effective formation of the full heterotrimeric Gαβ_1_γ_9_ complex than Gαβ_1_γ_2_. It was previously reported that some combinations of Gα and Gβγ were less stable and dissociated over longer periods of time [28]. On the other hand, the coupling of the Gαβ_1_γ_2_ protein to dopamine D_2_R or adenosine A_2A_R, in response to agonist activation, may be more productive than interaction with Gαβ_1_γ_9_. In cryogenic electron microscopy (cryo-EM) structures, there are no visible interactions between the Gγ subunit and A_2A_R [29] or with other receptors like 5-HT1B [29], A_1_R [30] or μOR [31]. Nonetheless, the subclass of the Gγ subunit is essential in governing the formation of a GPCR–G-protein complex, as it was reported for NTR1, adenosine A1 and muscarinic M2 receptors [28,32,33]. It has been shown that, especially, the C-terminal amino acid sequence of the Gγ subunit and its native acyl group are important determinants for the interaction between GPCR and the G-protein heterotrimer [33].

### 3.2. Analysis of G-Protein Interactions in Live Cells Using FLIM–FRET Microscopy

FLIM–FRET was employed to assess whether the Gγ subunits played a role in plasma membrane localization and formation of the G-protein heterotrimer. For these reasons, we performed experiments to evaluate the molecular interactions of two dimers, Gβ_1_γ_2_ and Gβ_1_γ_9_, with Gαs and Gαi_3_. As we have reported, these two different Gα subclasses show different lipid preferences and, consequently, are localized in distinct types of membrane domains [25]. Thus, we examined here if heterotrimer formation was also controlled in a Gα-subclass dependent manner.

**Figure 2:**
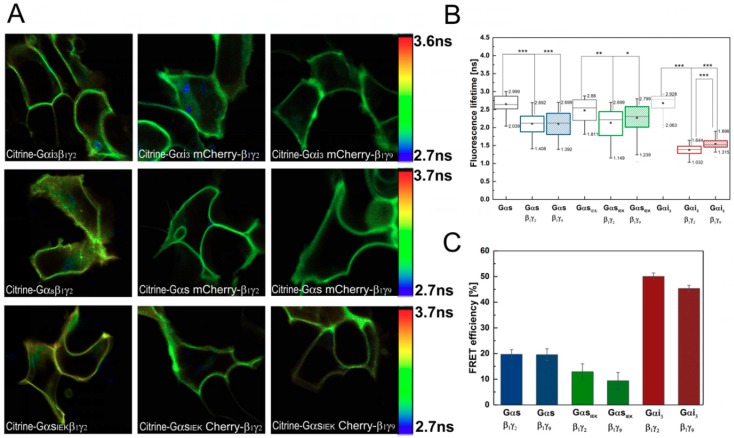
FLIM–FRET of Gαsβ_1_γ_2_, Gαsβ_1_γ_9,_ Gαi_3_β_1_γ_2_, and Gαi_3_β_1_γ_9_ heterotrimers. (**A**) HEK 293 cells were transiently transfected with Citrine–Gα alone or both mCherry–Gβγ and Citrine–Gα. Fluorescence lifetimes are presented in a continuous pseudo-color scale representing time values ranging from 2.7 (blue) to 3.6 (red) or 3.7 ns (red). (**B**) Box-and-whisker plots of the fluorescence lifetime *τ*_1_ of energy donor (Citrine–Gα) and donor in the presence of acceptor (mCherry–Gβγ). The median is shown as a line in the box, while the bottom and top boundaries represent the lower and upper quartile, respectively. Statistical significance of the difference in the donor fluorescence lifetimes *τ*_1_ detected in the absence and presence of energy acceptor using Mann–Whitney *U* test (* *p* < 0.02, ** *p* < 0.002, *** *p* < 0.0002). Gαs n = 42; Gαs and Gβ_1_γ_2_ n = 71; Gαs Gβ_1_γ_9_ n = 46; Gαi_3,_ and Gβ_1_γ_2_ n = 64; Gαi_3_ and Gβ_1_γ_9_ n = 72; Gαs_IEK_ and Gβ_1_γ_2_ n = 42; Gαs_IEK_ and Gβ_1_γ_9_ n = 38. (**C**) Plot of calculated fluorescence resonance energy transfer (FRET) efficiency percentage *E* derived from *τ*_1_, error bars represent standard errors.

We monitored FRET by measuring the lifetime of the donor fluorophore (Citrine) in the absence and presence of the acceptor (mCherry), as described in the Materials and Methods section. In our experimental system, Citrine (Citrine–Gαi_3_β_1_γ_2_, Citrine–Gαsβ_1_γ_2_) exhibited a double exponential decay with lifetimes of 2.7 ± 0.1 ns (*τ*_1_) and 3.4 ± 0.15 ns (*τ*_2_), implying the existence of two donor species. The amplitude of each of these lifetimes was approximately 50%. The presence of multi-exponential fluorescence decays in various intrinsically FP such as Citrine, cyan fluorescent protein (CFP), enhanced cyan fluorescent protein (ECFP), monomeric green fluorescent protein (mGFP), and Discosoma red fluorescent protein (DsRed) has been reported in several articles [34,35,36]. In the FRET system (the cells expressing Citrine–Gα were additionally transfected with mCherry–Gβ_1_ and the appropriate Gγ), the donor emission curves were also fitted to a double exponential decay model. However, a shortening of the fluorescence lifetime due to FRET was observed only for the short component *τ*_1_, while *τ*_2_ remained almost unchanged (compared to the donor alone). This indicates that only one donor species (characterized by lifetime *τ*_1_) underwent FRET (FRETing donor fluorophore state) and that the other species with the longer lifetime (*τ*_2_) was not engaged in FRET (non-FRETing donor fluorophore state). Therefore, only the FRETing component was used to calculate FRET efficiency (Figure 2C). Lifetime shortening due to FRET was also observed in the FLIM images as a uniform change in color toward the blue hues across all pixels (Figure 2A).

As shown in Figure 2B, different combinations of Gα subunits and Gβ_1_γ dimers gave varying levels of donor lifetime changes (box chart), and thus unequal FRET efficiencies. The highest FRET efficiency of 50% was obtained in cells expressing Gαi_3_β_1_γ_2_ and the efficiency was only 5% lower for the Gαi_3_β_1_γ_9_ heterotrimer. In contrast to Citrine–Gαi_3_, Citrine–Gαs exhibited a lower FRET signal when paired with either mCherry–Gβ_1_γ_2_ or mCherry–Gβ_1_γ_9_. The lifetime of Citrine–Gαs in cells co-expressing the mCherry–Gβ_1_γ_2_ dimer was found to be 2.12 ± 0.21 ns, amounting to 19.7% energy transfer efficiency, and a similar value was obtained for the complex with the mCherry–Gβ_1_γ_9_ dimer, giving a FRET signal of 19.5%.

**Figure 3:**
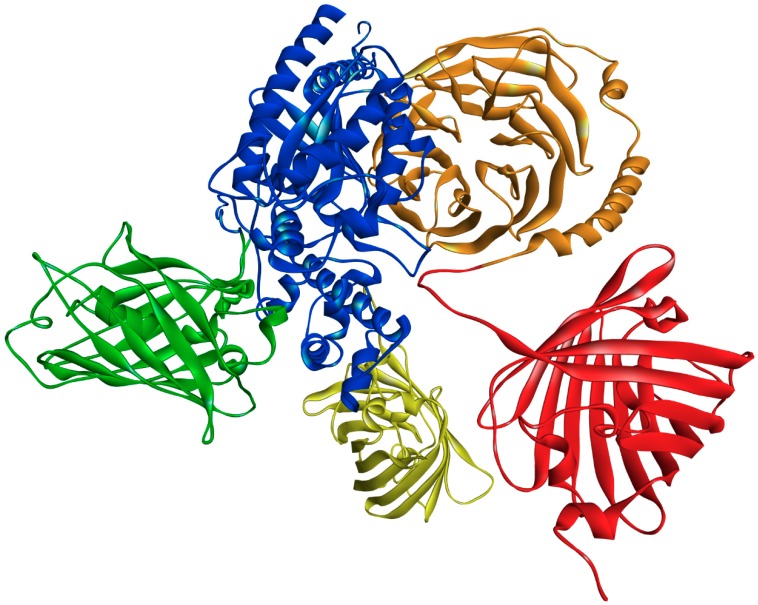
Localization of FP in the structures of Gαs and Gαi_3_. The molecular models of Citrine (green)–Gαs (blue), Citrine (yellow)–Gαi_3_ (concealed), and mCherry (red)–Gβ_1_ (orange) were generated with Chimera 1.13.1 [37] based on the structures of FP, Gαs, Gαi_3_, and Gβ_1_ (PDB id.: 1xa9, 6crk, 2ode, 6b3j, respectively). The models generated for fusion proteins were visually inspected, and the best-scored structures with suitable loops were chosen. The structure of a heterotrimeric complex of the investigated fusion proteins was recreated with the Discovery Studio software, version 4.0 (BIOvIA, D. S., San Diego, CA, USA, 2015).

An important question is whether the differences in FRET efficiency we observed are a measure of the efficiency of association between the interacting proteins or a consequence of structural differences between the heterotrimers. In the case of two proteins labeled with donor and acceptor molecules, FRET is highly dependent not only on the distance between the donor and acceptor but also on the stoichiometry of macromolecular interactions as well as on the donor fraction taking part in complex formation with acceptors. Therefore, an increase in FRET efficiency in our system can be interpreted as a quantitatively higher percentage of complexes formed between a particular Gα and Gβγ, especially when we are comparing trimers with the same Gα. Since Citrine in fusion proteins is integrated into other loops in the Gαs and Gαi_3_ structure (Figure 3), the structural differences between their heterotrimers should also be considered.

**Figure 4:**
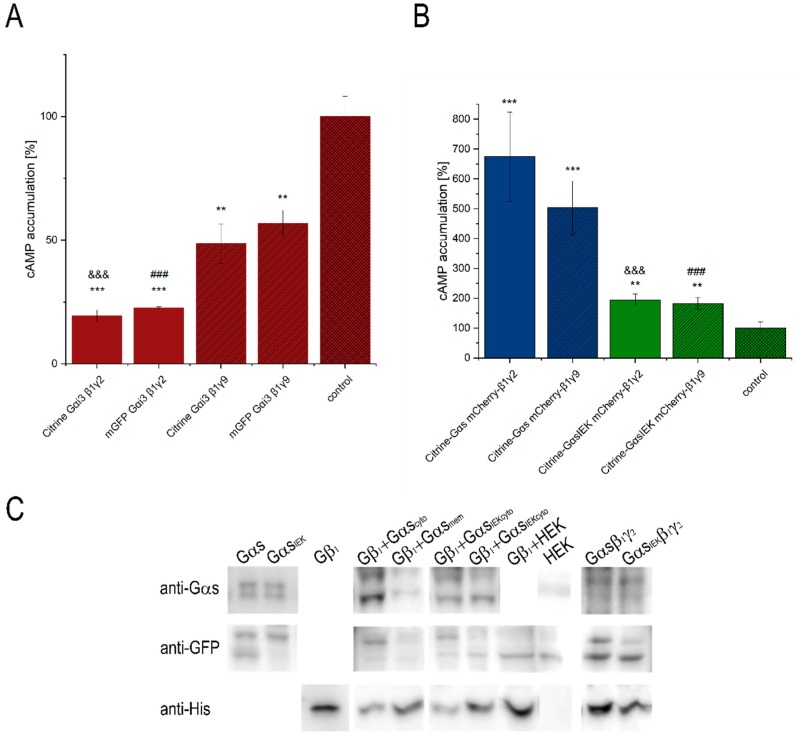
Effect of different Gγ subunits on the production of cAMP. HEK 293 cells were transiently transfected with Citrine–Gαi_3_, dopamine receptor D_2_R, mCherry–Gβ_1_γ_2_/γ_9,_ or Citrine–Gαs/s_IEK_, adenosine receptor A_2A_R and mCherry–Gβ_1_γ_2_/γ_9_. The activity of the investigated proteins after stimulation of the D_2_ receptor with sumanirole maleate or the A_2A_ receptor with 2-phenylaminoadenosine was determined by measurements of cAMP levels. Data are presented as percentage of mean cAMP level in control (non-transfected cells) which are considered as intrinsic cAMP levels after stimulation of the D_2_ or A_2A_ receptor. Differences in cAMP levels between samples were evaluated by the unpaired *t*-test. (**A**) Citrine–Gαi_3_: comparison with the control: **-*p* < 0.01 ***-*p* < 0.001, Citrine–Gαi_3_ mCherry–Gβ_1_γ_2_ vs. Citrine–Gαi_3_ mCherry–Gβ_1_γ_2_ &&&-*p* < 0.001 and mGFP–Gαi_3_ mCherry–Gβ_1_γ_2_ vs. mGFP–Gαi_3_ mCherry–Gβ_1_γ_9_ ###-*p* < 0.001 (n = 8). (**B**) Citrine–Gαs/s_IEK_: comparison with the control: **-*p* < 0.01 ***-*p* < 0.005, Citrine–Gαs mCherry–Gβ_1_γ_2_ vs. Citrine–Gαs_IEK_ mCherry–Gβ_1_γ_2_ &&&-*p* < 0.01, and Citrine–Gαs mCherry–Gβ_1_γ_9_ vs. Citrine–Gαs_IEK_ mCherry–Gβ_1_γ_9_ ###-*p* < 0.005 (n = 8). (**C**) In a pull-down assay, Gαs_IEK_ was found to interact with Gβ_1_γ_2_. Recombinant His-tagged mCherry–Gβ_1_ bound to NiNTA beads were incubated with Citrine–Gαs or Citrine–Gαs_IEK_ cell lysates. Beads were precipitated, and the amount of Gα was detected by Western blotting, using an antibody specific to Gαs. The anti-His-tag antibody was used for visualization of His-tagged mCherry–Gβ_1_. The figure is representative of three independent experiments.

Considering the results of intracellular cAMP concentrations obtained for Gαi_3_ heterotrimers, it seems reasonable to conclude that FRET efficiency for these complexes may be related to their content at the plasma membrane. The smaller FRET efficiency between Citrine–Gαi_3_ and mCherry–Gβ_1_γ_9_ is reflected in a smaller decrease in cAMP concentration when compared to Gαi_3_β_1_γ_2_. No differences were found in FRET efficiency between the investigated Gαs heterotrimers, indicating a similar level of Gαsβ_1_γ_2_ and Gαsβ_1_γ_9_ complexes at the plasma membrane. However, as shown in Figure 4B, a slight difference in the cAMP concentrations of Gαsβ_1_γ_2_ and Gαsβ_1_γ_9_ can be observed, although it is not statistically significant and is smaller than between Gαi_3_ heterotrimers. Thus, even if there is a difference in the concentrations of these complexes at the plasma membrane, it is not substantial. On the contrary, there are significant differences in FRET signals between Gαs and Gαi_3_ heterotrimers which cannot be uniquely attributed to only a single factor. Most probably the FRET signal reflects a mixture of effects, i.e., a potentially lower percentage of trimers composed of Gαs and Gβ_1_γ_2_ or Gβ_1_γ_9_ dimers than Gαi_3_ heterotrimers, and structural differences between these heterotrimers due to different localizations of fluorescence donors (Figure 3). The estimated difference in the distance separating mCherry–Gβ_1_ and Citrine–Gαs is, on average, 6.5 Å longer than in the Gαi_3_ heterotrimer. Thus, the energy transfer efficiency in the Gαs heterotrimers should be lower by about 7% than in Gαi_3_ heterotrimers, assuming Förster distance R_0_ of 56.6 Å for this pair of fluorophores [38,39].

As all the investigated Gα subunits and Gβγ dimers exist as complexes and interact directly in our FRET-FLIM assays (Figure 2A–C), we further examined the sensitivity and specificity of the measured FRET signals. In order to improve the detection sensitivity of FRET, we treated the cells with GppNHp (5′-guanylimidodiphosphate; concentration: 0.1 or 0.2 mM; time frame: 0–60 min.), a non-hydrolysable analog of GTP, to induce subsequent dissociation of the Gα–GppNHp complex from Gβγ. Since the G-protein is continuously active, the FRET signal between Citrine–Gα and mCherry-Gβ_1_ was expected to be significantly reduced; however, no such changes were observed for any of the examined heterotrimers. We also tested GppNHp at an eight-fold higher concentration and again observed insensitivity or occasional reduction of the FRET signal. This suggests that the heterotrimer was not affected by GppNHp added to the cell culture. These findings prompted us to construct a mutant of Gαs, defective in the formation of functional heterotrimers rather than in the permeabilization of cell membranes, leading to a disruption of the native membrane. The residue Ile_26_–Glu_27_–Lys_28_ of Gαs has long been recognized as being essential for its interaction with Gβ, and a suitable mutation is believed to impair the assembly of a functional heterotrimer [40]. We engineered a Gαs_IEK_ mutant in which each of the interacting amino acid residues was substituted with alanine and expected that the formation of the heterotrimer would be impaired. Similar mutation constructs were previously shown to have a strong propensity to disrupt membrane localization and palmitoylation of the Gαs subunit as well as deficient binding to Gβγ [23]. To examine the effect of the IEK mutation on the ability of Gαs to interact with Gβγ, Gαs_IEK_ was co-expressed with Gβ_1_γ_2_ or Gβ_1_γ_9_ in HEK 293 cells. As shown in Figure 1, Gαs_IEK_ exhibited plasma membrane localization and co-localization with Gβ_1_γ_2_ or Gβ_1_γ_9_, but to a lower extent than the wild-type Gαs. We also examined cAMP production to ensure that the mutation introduced in Gαs fulfilled its role. As shown in Figure 4B, a clear reduction in cAMP production was observed as compared to the wild-type Gαs and the cAMP concentration was approximately three times lower than for Gαs. These findings suggest a lower Gαs_IEK_ heterotrimer content than that of Gαs at the plasma membrane, which led to a reduced activation of adenylyl cyclase.

To further confirm that the observed increase in cAMP concentration over the basal level resulted from the direct interaction of Gβ_1_ with Gαs_IEK_, we performed a pull-down assay. Cell lysates containing Citrine–Gαs_IEK_ or Citrine–Gαs were loaded onto NiNTA agarose baited with His_6_-tagged forms of mCherry–Gβ_1_ and then the complexes were eluted. Furthermore, purified proteins were detected by immunoblotting with an antibody against the N-terminal epitope of Gαs and against the His_6_-tag of Gβ_1_. Figure 4C summarizes the results of all pull-down experiments, showing the binding of Gαs_IEK_ to His_6_-tagged Gβ_1_. No clear signal was observed for the negative cell lysate (Figure 4C), thus confirming that the band detected by the anti-Gαs antibody is Gαs_IEK_ associated with Gβ_1_ and not endogenous Gαs present in the cell lysate. Incubation of the membrane fraction of cells overexpressing Gαs_IEK_ and Gβ_1_γ_2_ with the NiNTA resin resulted in a signal from Gαs_IEK_, once again indicating the presence of the full Gαs_IEK_β_1_γ_2_ heterotrimer. Since these pull-down experiments are qualitative or at best semi-quantitative in nature, we did not attempt to quantify the relative amounts of the Gαs_IEK_β_1_γ_2_ and Gαsβ_1_γ_2_ heterotrimers.

Our data strongly suggest that the IEK mutation in Gαs is Gβγ binding-deficient, the heterotrimer is formed less efficiently and has impaired activity but is still able to activate adenylyl cyclase. The examined Gβ-binding surface on the Gα subunit may not be the only essential interacting region of the heterotrimer assembly. The mutation did not prevent the heterotrimer formation but confirmed the specificity and sensitivity of the measured FRET signals. The FRET efficiency between Citrine–Gαs_IEK_ and mCherry–Gβγ dimers was reduced to 12.9% and 9.4% as compared to wild-type Gαs in the presence of Gβ_1_γ_2_ and Gβ_1_γ_9_ dimers, respectively (Figure 2C). These results demonstrate that differences in the heterotrimer levels of the mutant Gαs_IEK_ and the wild-type protein are reflected in the detection of a lower FRET signal and are also consistent with the results of intracellular cAMP concentration measurements. Overall, this study confirmed the sensitivity and accuracy of FLIM–FRET as a suitable tool for studying the interactions of signaling proteins.

### 3.3. Effect of Gβγ Dimer on Gα Diffusion

As stated earlier, the apparent diffusion coefficients of full heterotrimers differ significantly from those of monomers of Gα subunits. The formation of the complex of the Gα subunit with the Gβγ dimer modulates the mobility of the full heterotrimer, while the presence of a specific receptor is significant as well. Here, the presence of the Gβ_1_γ_9_ dimer caused an increase in apparent diffusion coefficients for all observed Gα subunits but in a different manner (Table 1). In the case of co-expression of the Gαi_3_ subunit with the Gβ_1_γ_9_ dimer, the diffusion coefficient was 0.475 μm^2^/s, and was significantly higher compared to the value of 0.424 μm^2^/s obtained with the Gβ_1_γ_2_ dimer (Table 1). Conversely, for the Gαs subunit, the measured value of apparent diffusion coefficient in the presence of the Gβ_1_γ_9_ dimer was 0.202 μm^2^/s and was higher than for the Gαs subunit itself (0.130 μm^2^/s, Table 1), but significantly lower than the value measured in the presence of the Gβ_1_γ_2_ dimer–0.246 μm^2^/s (Table 1).

For the modified Gαs_IEK_ subunit, the apparent diffusion coefficient obtained in the presence of the Gβ_1_γ_2_ or Gβ_1_γ_9_ dimers was the same (0.214 μm^2^/s). The modification of the Gαs_IEK_ subunit did not affect the diffusion of this trimer subunit with the Gβ_1_γ_9_ dimer and the difference was statistically insignificant. However, the diffusion rate was reduced significantly for the subunit Gαs_IEK_ in the presence of the Gβ_1_γ_2_ dimer in comparison to the Gαsβ_1_γ_2_ heterotrimer (0.246 μm^2^/s, Table 1). This may indicate a selective character of mutations within the region of interaction of the Gαs subunit with the Gβγ dimer. On the other hand, the modified Gαs_IEK_ subunit was less effective in creating complexes with a Gβγ dimer (lower FRET efficiency). The diffusion coefficient observed may, therefore, be reduced by the presence of a monomeric subunit at the cell membrane.

## 4. Discussion

In this study, we have combined two live-cell imaging microscopic methods, FLIM–FRET and FRAP, to investigate the molecular-level assembly properties and the trafficking dynamics of G-protein heterotrimers within the cell membrane. The membrane dynamics of heterotrimer complexes were monitored by FRAP. The studied Gαs and Gαi_3_ subunits are expressed in most types of cells. Similarly, the Gβ_1_ and Gγ_2_ subunit expression is ubiquitous, whereas Gγ_9_ is mostly present in olfactory epithelium [1].

Although much progress has been made in understanding the molecular details of how G-proteins interact with GPCRs and regulate the activity of their downstream targets, it is less clear how activated GPCRs initiate this process and what the trafficking pathway of the heterotrimeric G-proteins is within the plasma membrane. The role of the plasma membrane lateral organization in the spatiotemporal distribution of GPCRs and G-proteins appears to be essential in the process of extracellular signal transduction. However, the molecular basis for the interaction of signaling molecules with lipids is still not fully understood but seems to be of key importance in understanding the functional selectivity and activation speed of cellular responses to G-protein activation. Previous evidence supports the direct role of the Gγ subunit in G-protein activation by a receptor [41,42,43], and also suggests Gγ diversity as a crucial modulator of G-protein membrane localization behaviors as well as trimer assembly [44,45,46]. Using the same FLIM–FRET and FRAP approach, we previously reported that preferences in localization within the membrane of the stimulatory and inhibitory Gα subunits, Gαs and Gαi_3_, are different and further modulated by the dopamine D_1_ receptor, as well as by the Gβ_1_γ_2_ dimer [22,25]. The association of Gβ_1_γ_2_ with the GDP-bound Gα subunit translocates G-proteins outside the liquid-ordered membrane domains, which is particularly evident for Gαs [25]. Here we confirmed the ability of the Gγ subunit to bind specific lipids, and consequently, to influence the membrane localization of the full heterotrimeric complex of the G-protein; but our results further showed that its membrane distribution was not only Gγ-subclass dependent.

In this study, we examined the impact of two distinct Gγ subunits, Gγ_2,_ and Gγ_9_, on the membrane localization of G-proteins. These Gγ differ, among others, in membrane anchors at carboxyl-terminal cysteine in the CaaX motif. The prenyl group promotes tethering of Gβγ complexes to membranes, sorting of particular lipid domains, as well as playing a role in the translocation properties of the Gβγ dimer and effector activation [47,48]. The Gγ_2_ belongs to the group of slowest translocating Gγ subunits (t_1/2_ ~130 s) whereas Gγ_9_ belongs to the fastest one (t_1/2_ ~ 10 s) [19,20]. The prenylation of Gγ_2_ with a 20-carbon geranylgeranyl lipid, together with positively charged residues in the C-terminal domain, provides it with a higher affinity for the plasma membrane than Gγ_9_ with the 15-carbon farnesyl lipid attachment and fewer positively charged residues. The presence of a five-residue or six-residue cluster of positively charged amino acids in the pre-CaaX region modulates Gγ–membrane interactions, strengthening the plasma membrane affinity [20], and also governs the membrane-interacting ability of Gβγ [42]. Considering how many possible Gβγ and Gα combinations exist, a key question in G-protein signaling is whether the plasma membrane location of heterotrimers composed of distinct subunits is only Gβγ-dependent. Indeed, our FRAP data for Gγ subunits correlated well with translocation rates in a Gγ-dependent manner only for heterotrimers composed of Gαi_3_. The mobility of Gαi_3_β_1_γ_9_ is much higher than that of Gαi_3_β_1_γ_2_ and its population in the membrane is also lower. On the contrary, Gβ_1_γ_2_ associated with Gαs diffuses faster than Gαsβ_1_γ_9._ In fact, both Gαsβγ heterotrimers diffused significantly slower when compared with their Gαi_3_ associates. At the same time, if the concentrations of both the Gαs heterotrimers at the plasma membrane are comparable, the reduced mobility of Gαsβ_1_γ_9_ cannot be considered to be the result of a higher proportion of uncomplexed Gαs. The diffusion data thus indicated that the membrane localization of G-proteins was dependent not only on Gβγ but also on the Gα subtype since distinct heterotrimeric combinations showed different mobility characteristics.

The Gα subunits are palmitoylated and mostly myristoylated, depending on the specific Gα-subclass. The dual, N-palmitoylation and S-palmitoylation, of Gαs is similar to the N-myristoylation and S-palmitoylation motif of the Gαi class, but they differ in the number of positive charge residues at the N-terminus [6,49]. The membrane binding area of Gαs or Gαi_3_ is limited to two sites on the surface of the protein and the membrane [50]. However, most of the membrane binding area of Gα is formed by the N-terminus with covalently attached lipids. Consequently, since the IEK mutation reduces palmitoylation, apart from disrupting Gβ_1_ coupling, it also affects proper membrane localization of Gαs. Since this mutation reduces palmitoylation, it affects the specific binding of Gαs to the plasma membrane [23]. Indeed, our diffusion data suggest that the N-termini residues of Gαs function as an essential signal to ensure the correct localization of the subunit at the plasma membrane. Unlike for the heterotrimers formed by the wild-type Gαs and Gαi_3_, the diffusion coefficient of distinct Gαs_IEK_β_1_γ_2_ and Gαs_IEK_β_1_γ_9_ heterotrimers is equal, as is the intracellular cAMP concentration, indicating that the mutation eliminates the specificity of the Gαs_IEK_ heterotrimers. However, it is difficult to clearly indicate whether the substitution of charged residues in the Gβγ interacting surface or the reduced palmitoylation impairs the specificity of heterotrimer membrane targeting. Nevertheless, the presented results support the notion of the presence of membrane attachment signals in the N-termini of Gα subunits.

As we previously reported, preferences in localization within the membrane of the stimulatory and inhibitory Gα subunits are different [25]. Gαs prefers solid-like domains (insensitive to cholesterol and structure or composition of lipid rafts), while Gαi_3_ prefers the more fluid regions of the membrane and also detergent-resistant lipid rafts. The membrane mobility of Gαs is relatively slow, while the Gαi_3_ diffusion is much faster. When Gβγ dimer binds to the Gα, despite the increase in the molecular weight of the complex, it accelerates the lateral diffusion of Gα in all tested heterotrimers. Thus, this finding again strengthens the hypothesis that the Gβγ dimer not only affects the diffusion of Gα but also relocates complexed Gα within the plasma membrane. Notably, it is evident in the case of Gαs heterotrimers because the apparent diffusion coefficient is more than 1.5 times greater than for the uncomplexed subunit. As demonstrated previously, the Gβγ dimer is responsible for the rapid relocation of Gα from the lamellar membrane region where it resides as a monomer [25,51]. The Gβγ complex remains associated with the non-lamellar regions which may explain the acceleration of the diffusion rate of heterotrimers compared to monomeric Gα [51]. Interestingly, as mentioned above, the lateral mobility of the G-proteins composed of the Gβ_1_γ_2_ dimer differs significantly from those containing Gβ_1_γ_9_ dimer; the diffusion rates of distinct heterotrimers are characterized by different values of diffusion coefficients. Hence, while the Gβγ dimer defines the affinity of the complete heterotrimer for the lipid phase, the differences in the prenyl moieties are not sufficient to explain the differential diffusion of the full heterotrimers. Our data correspond with the FRET-clustering analysis of fluorescently-tagged heterotrimeric G-protein-derived membrane anchors [52]. The authors have shown that the N-terminal sequences of Gαi/o and Gαq and their heterotrimers (the N-terminal part of Gαi_2_ or Gαq merging with the C-terminal part of Gγ_2_) were clustered together in different domains. Moreover, postulated membrane domains partially share their area causing overlapping domains, thus strengthening co-clustering. Upon activation the heterotrimers dissociate and the Gα subunits displace into a subclass-specific domains. Our FRAP data do not support the pre-activation co-clustering of the distinct heterotrimeric G-proteins. A possible explanation for this discrepancy in conclusions correlates with the use of membrane anchors of Gα and Gγ subunits but not, as in the present study, with full-length G-proteins.

In addition to those previously identified, both components, Gβγ dimer and Gα, determine final membrane localization of the full heterotrimer. These findings imply that the dissociation of the G-protein on activation and subsequent re-association on deactivation, are also influenced by the subclass of the Gα subunit. Yet, the FRAP measurements could not precisely resolve membrane distribution of G-protein but strongly suggested the possibility of different localizations at the plasma membrane of particular heterotrimers (membrane domains/regions differing in lipid composition and properties). The results presented here indicate that the diffusion rates of heterotrimers composed of different Gβγ and Gα subunits were not directly related to the membrane dissociation Gγ-pattern that were determined by the nature of the prenyl group and by basic residues in the C termini of Gγ. The role of the Gα subunit in determining the membrane localization by interacting with lipids and Gβγ dimers shown in the present study suggests that the Gγ translocation rate can, consequently, be also affected by the Gα subunit.

The mobility of the G-proteins and dissociated subunits is heterogeneous, suggesting non-random distribution within the cell membranes, which may strongly reflect the natural functions of these proteins. Many studies have emphasized the importance of the clustering of membrane proteins in a manner dependent on their functional state [53,54]. Results obtained in the present study show that divergent heterotrimers localize to distinct membrane locations due to the combined lipid modifications on Gα and Gγ, together with a different number and distribution of adjacent positively charged residues (i.e., various classes of G-proteins located in distinct domains relocate upon activation and dissociation into subunits). Since the cell membrane is currently considered as a highly complex structure, the role of distinct types of membrane domains in the spatiotemporal organization of GPCRs and G-proteins in the process of signal transduction needs further studies.

## Figures and Tables

**Table 1:** Lateral diffusion coefficients of investigated Gα subunits fusion protein constructs in the presence of Gβ_1_γ_2_ or Gβ_1_γ_9_ dimers.

	D_app_ (µm^2^/s)	Mf (%)	n
Gαs ^†^	0.130 ± 0.004	84.5 ± 1.5	49
Gαs Gβ_1_γ_2_	0.246 ± 0.009	92.4 ± 0.8	143
Gαs Gβ_1_γ_9_	0.202 ± 0.007	89.5 ± 1.1	92
Gαi_3_^†^	0.338 ± 0.022	94.2 ± 1.7	34
Gαi_3_ Gβ_1_γ_2_	0.424 ± 0.014	93.5 ± 0.9	66
Gαi_3_ Gβ_1_γ_9_	0.475 ± 0.021	92.8 ± 1.2	60
Gαs_IEK_ Gβ_1_γ_2_	0.214 ± 0.010	87.8 ± 1.5	61
Gαs_IEK_ Gβ_1_γ_9_	0.214 ± 0.005	89.7 ± 0.9	108

In the experiments where the co-expression took place, the diffusion of Gα subunits was measured. Values represent the mean ± S.E.M., D_app_—apparent diffusion coefficient, Mf—mobile fraction, ^†^—data from Reference [22].
